# Delirium misdiagnosis risk in psychiatry: a machine learning-logistic regression predictive algorithm

**DOI:** 10.1186/s12913-020-5005-1

**Published:** 2020-02-27

**Authors:** Catherine Hercus, Abdul-Rahman Hudaib

**Affiliations:** 1 Alfred Hospital, Melbourne, Australia; 2 Monash Alfred Psychiatry Research Centre (MAPRc), Melbourne, Australia

**Keywords:** Delirium, Misdiagnosis, Machine learning-logistic classifier, Input variables

## Abstract

**Background:**

Delirium is a frequent diagnosis made by Consultation-Liaison Psychiatry (CLP). Numerous studies have demonstrated misdiagnosis prior to referral to CLP. Few studies have considered the factors underlying misdiagnosis using multivariate approaches.

**Objectives:**

To determine the number of cases referred to CLP, which are misdiagnosed at time of referral, to build an accurate predictive classifier algorithm, using input variables related to delirium misdiagnosis.

**Method:**

A retrospective observational study was conducted at Alfred Hospital in Melbourne, collecting data from a record of all patients seen by CLP for a period of 5 months. Data was collected pertaining to putative factors underlying misdiagnosis**. A** Machine Learning-Logistic Regression classifier model was built, to classify cases of accurate delirium diagnosis vs. misdiagnosis.

**Results:**

Thirty five of 74 new cases referred were misdiagnosed. The proposed predictive algorithm achieved a mean Receiver Operating Characteristic (ROC) Area under the curve (AUC) of 79%, an average 72% classification accuracy, 77% sensitivity and 67% specificity.

CONCLUSIONS*:* Delirium is commonly misdiagnosed in hospital settings. Our findings support the potential application of Machine Leaning-logistic predictive classifier in health care settings.

## Background

Delirium is a common neuropsychiatric syndrome, occurring in 14–56% of elderly inpatients, with rates up to 82% in ventilated patients [1–3]. Delirium is associated with higher morbidity, mortality, length of stay and readmission rates, functional decline, and poorer quality of life, greater risk of long-term institutional care and increased health and social care expenditure [4–6]. It is a clinical diagnosis, made based on acutely disturbed attention and impaired cognition [6]. DSM but not ICD criteria additionally require one or more underlying etiological factors, although these can be challenging to demonstrate [7].

Delirium is frequently seen by Consultation Liaison Psychiatry (CLP), with rates varying from 4% [8] to 39% [9] of CLP referrals. Despite its high prevalence, misdiagnosis of delirium prior to CLP referral is common; various factors underlying this phenomenon have been described [3, 6, 7, 10–13].

Rates of delirium misdiagnosis range in the majority of studies from 41.8% [11] to 64% [3, 6, 7, 10–13]. Otani et al. found a low misdiagnosis rate of 8.1%, and related this to a relatively better understanding of psychiatric diagnosis by physicians in their hospital [14]. In contrast, Joshi et al. found a much higher misdiagnosis rate of 73%, relating to their stringent classification as inaccurate in all cases where delirium was not mentioned specifically in the referral, even if delirium diagnosis was documented in the patient file [15]. In non-CLP settings, similar rates have been observed, with 61% of delirium misdiagnosed by the primary team prior to referral to a palliative care inpatient service [13]. The wide variation in delirium misdiagnosis rates may reflect variation in study design and setting and limitations in study sample size.

Various patient, clinician and system factors have been associated with misdiagnosis of delirium [5]. Patient factors suggested to increase rates of delirium misdiagnosis include age, pain, sex, psychiatric comorbidity and hypoactive delirium subtype. Direct evidence and consensus regarding these associations varies. Increasing age is a risk factor for increased delirium incidence. However, elevated rates of delirium misdiagnosis have been reported in both older [14] and younger [3, 16] patients. Further, other studies have found no association [10, 13].Likewise pain is widely acknowledged as an aetiological factor for delirium, but may also make misdiagnosis more likely, with signs of delirium attributed to pain. Indeed, patients with pain were more likely to be misdiagnosed in both CLP [10] and palliative care settings [13]. While sex was not shown by Swigart et al., nor Kishi et al., to predict misdiagnosis [3, 10], Nicholas et al. found that patients referred with a diagnosis of depression later found to have delirium, were significantly more likely to be male than female [7].

A past psychiatric history has been shown in two studies to make delirium misdiagnosis more likely [10, 15], though not in a palliative care setting [13]. In another study, Swigart et al. did not find that an overall psychiatric diagnosis made misdiagnosis more likely, but specific diagnoses – bipolar disorder and psychosis – conferred nine times greater odds of misdiagnosis [3].

Hypoactive delirium is more likely to be misdiagnosed [6, 17], as vegetative symptoms usually associated with depression or medical illness are prominent and attract less concern than agitated hyperactive symptoms [6, 11]. Indeed, Mittal et alshowed that hypoactive delirium patients are less likely to be referred to psychiatry than patients with a hyperactive or mixed type [18]. A study examining older patients in emergency departments found that 78.3% of hypoactive delirium was not recognized by emergency physicians [19]; failure to recognize hypoactive delirium was also noted by Meagher and Trzepacz [20], and Inouye et al. [21].

Other factors affecting rates of delirium misdiagnosis include knowledge and training of clinicians and patient setting. For example, while medical specialists outperformed family practice physicians and surgeons in a survey on organic mental disorders [22], family practice physicians were more likely to accurately diagnose delirium [3]. In contrast, Farrell and Ganzini found no difference in accuracy of diagnosis between specialties, or between providers with different levels of training (i.e. nurses, residents, or staff physicians) [11].

Delirium diagnosis is aided by the use of validated screening instruments such as the CAM-ICU and 4AT [23, 24] yet rates of use of validated screening instruments are low. Patel et al. found 59% of respondents reported regularly screening for delirium, but two-thirds of these did not use a validated instrument [25].

There are wide implications of misdiagnosis given the demonstrated impact of delirium on length of hospital stay, cost and ultimately, patients’ prognoses [2, 26]. Understanding the reasons that delirium is missed is therefore crucial. Previous studies of the factors underlying delirium misdiagnosis are limited due to analysis with underpowered multivariate logistic regression [3, 10, 15, 27].

This study aims to determine the number of CLP referrals diagnosed as having delirium over a 5-month period within our service, and the number of cases misdiagnosed on referral. Further, we use a Machine Learning classifier to predict accurate delirium diagnosis on referral.

## Method

Alfred Hospital ethics and research institutional board approved this study.

Patients were diagnosed with delirium based on assessment by a member of CLP staff (attending psychiatrist). Correct diagnoses were assigned if one of the differentials on referral was delirium, encephalopathy or post-traumatic amnesia.

Information pertaining to patients diagnosed with delirium was obtained from a CLP database, and individual patient medical records were searched electronically. The following information was recorded for each patient: age, sex, date referred, date admitted, date discharged or transferred, referral diagnosis, length of stay, referring team, past psychiatric history presence and diagnosis if relevant, pain as a primary symptom, whether or not delirium was hypoactive, whether 4AT was completed and score, and mortality.

### Predictive classifier model building

The number of input variables was nine. The Machine Learning- logistic regression model used all potential predictors from a prior theoretical framework. Logistic regression is a parametric maximum likelihood method for binary classification tasks where predicted probability for each category of the dependent variable is produced. Data was split into test (20% of the sample, performed with SPM software, Salford Systems) and training datasets, however, for the purpose of brevity, the performance metrics for the classifier from the training dataset is listed. To formally evaluate the model’s overall predictive efficiency we reported mean accuracy metrics (sensitivity, specificity, AUC and its 95% CI, classification accuracy). Further, we assessed model fitness (Hosmer-Lemeshow statistic). All models were written with Salford Systems (SPM (ver. 8.3.0), San Diego, California, USA).

## Results

### Sample details/input variables

Over the study period, there were 584 new referrals to CLP. Of these, 74 were diagnosed with delirium. Number of patients with accurate diagnosis of delirium on referral was 39. There were two cases of delirium diagnosis with missing data across the nine input variables.

Patients diagnosed with delirium were identified from a source hospital database of all CLP referrals between November 2018 and March 2019. The patient population consisted of 31 females (43.1%) and 41 males (56.9%). The mean age was 61.2 (*SD* = 15.8), and the mean length of hospital stay (days) was 41.5 (*SD* = 41.2). Fourty-six patients were admitted to medical units, and 26 were surgical patients.

Patients admitted to medical wards were from general medicine (39%), burns (7%), oncology/hematology (8%), cardiology (6%), neurology (4%), and renal (3%), while, surgical patients were from general surgery (9%), neurosurgery (4%), trauma (12%), and lung transplant (7%).

Forty-four patients (61%) had history of psychiatric conditions (depression (39%), schizophrenia (23%), bipolar disorder (13%), dementia (12%), substance misuse disorder (7%), acquired brain injury (4%), and intellectual disability (2%)).

The odds ratios, as variable importance measures, and their corresponding 95% confidence intervals for the nine variables are listed in Table 1. Of note, a patient was nearly 5.5 times less likely to be accurately diagnosed with delirium if having a concurrent psychiatric history (*p* = 0.03). The odds ratios for cases with death outcome (0.52), hypoactive delirium subtype (0.58), age (0.97), having a 4-AT score filed before referral (4.16), female sex (3.35) and a referral from a medical unit (1.23), hospital stay duration(1.00) and labeled as patient with pain diagnosis (1.14).

**Table 1:** Machine Learning-Logistic regression classifier for accurate delirium diagnosis^a^

Variable	B (SE)	Odds ratio (95% CI)	P
Age	- 0.02(0.02)	0.97 (0.93 to 1.02)	0.29
Sex(Female)	- 1.21(0.71)	3.35 (0.83 to 13.42)	0.08
Referral unit(medical vs. surgical)	- 0.21(0.84)	1.23 (0.23 to 6.41)	0.79
Psychiatry history	**- 1.69(0.78)**	**0.18 (0.03 to 0.85)**	**0.03**
Pain^b^	- 0.13(0.83)	1.14 (0.22 to 5.88)	0.86
hypoactive delirium	- 0.53(0.84)	0.58 (0.11 to 3.03)	0.52
Death^c^	- 0.64(1.10)	0.52 (0.05 to 4.55)	0.55
Hospital stay(days)	- 0.0061(0.007)	1.00 (0.99 to 1.02)	0.40
4-AT^d^ score prior to referral (filed vs. non filed)	- 1.42 (0.84)	4.16 (0.80 to 21.45)	0.09

For the model: Hosmer-Lemeshow statistic = 10.28 (*p* = 0.24), *B* unexponentiated coefficient, *SE* Standard error

^a^number of patients with accurate diagnosis of delirium on referral was 39 (54%).

^b^Number of patients with referral diagnosis labelled as “pain” was 25

^c^Number of cases where 4-AT score was filed prior to referral was 41. Number of patients with hypoactive delirium subtype was 15

^d^7 patients with delirium diagnosis died during hospital admission

*Bold* some evidence against the null hypothesis of no association between psychiatric history and delirium diagnosis status, with odds ratio confidence interval not crossing the null

### Predictive model results

**Fig. 1:**
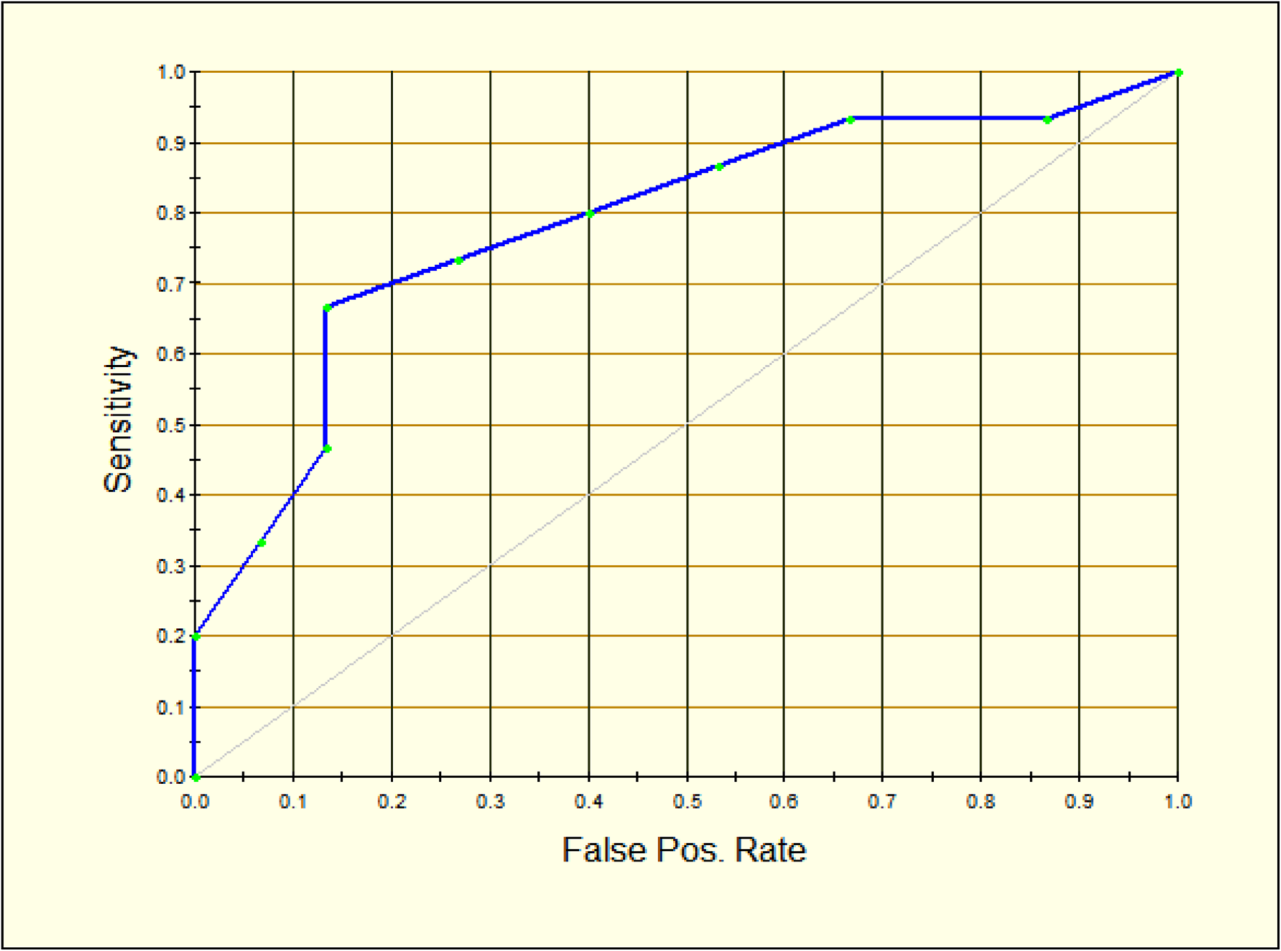
ROC curve representing the diagnostic accuracy of the proposed predictive classifier. As diagnostic test accuracy improves, the ROC curve moves toward the left and the AUC (Area under the curve) approaches 1

The performance of the predictive classifier model is reported in Table 2. The main accuracy metrics indicate the model shows higher sensitivity, specificity, negative predictive value (NPV), positive predictive value (PPV), and lower false positive and false negative rates. The mean ROC Area under the curve (AUC) is 0.79 (Fig. 1).

**Table 2:** Machine Learning-Logistic regression classifier performance

Metric	Predictive ability
Sensitivity (true positive rate)	0.77
Specificity (true negative rate)	0.67
False positive rate	0.33
False negative rate	0.23
Positive predictive value (PPV)	0.70
Negative predictive value (NPV)	0.74
AUC	0.79 (95% CI: 0.66 to 0.90)
Classification Accuracy	0.72

*AUC* Area under the curve

## Discussion

Using a Machine Learning-logistic classifier approach, this study explored and utilised the factors driving the common occurrence of delirium misdiagnosis on hospital wards. In a sample of consecutive CLP referrals in a major metropolitan hospital, we found a high number of misdiagnosed delirium cases; nearly half the patients referred to CLP were inaccurately diagnosed. The most important factor increasing the likelihood of misdiagnosis in the predictive model was a history of a psychiatric disorder. Other important input variables were hypoactive subtype of delirium, as were 4-AT score and female sex.

To our knowledge, this is the first study that has employed an unsupervised Machine Learning approach to the common clinical problem of delirium diagnosis. The advantage to using this approach is that it yields prediction accuracy performance in terms of area under the curve (AUC), sensitivity and specificity. The patient population in this study was largely reflective of a typical hospital setting in terms of mean age, variety of admitting clinical units and overall proportion of patients found to have delirium. Seventy-four of the 584 consecutive patients referred to CLP were due to suspected delirium, which corresponds to existing similar studies [14, 27]. The number of these patients that were misdiagnosed was 39 (53%) which is also in line with the existing literature [3, 10, 27]. Together, these figures reinforce the importance of addressing the issue of delirium misdiagnosis, as they show that delirium is a commonly occurring problem amongst hospital patients, and that it continues to be poorly identified by staff on the hospital wards. Knowing the serious clinical consequences of delirium, this speaks to the need for better education of medical and allied health staff in the area, and for research and development of clinical tools and strategies for aiding the accuracy of diagnosis in a patient with suspected delirium.

The strong association between the history of mental illness and inaccurate delirium diagnosis is consistent with previous studies [3, 10]. It highlights the issue of ‘diagnostic overshadowing’, which occurs when clinicians attribute common manifestations of delirium such as psychosis, changes in cognition and increased agitation to past psychiatric diagnoses [10]. In our study, 18 of 27, or two-thirds of patients with a history of mental illness who were referred to CLP with suspected delirium were misdiagnosed. This finding suggests that future education in general hospital settings should focus on distinguishing delirium from other psychiatric conditions such as depression, dementia and psychosis, and highlights the need for caution in interpreting symptoms as relating to past psychiatric diagnoses. The cognitive aspects of delirium, such as confusion and disorientation, are generally well recognized; it is the psychiatric features that are often the most striking, and show the most variation between cases. In delirium, any component of the mental state examination can be affected, including mood, affect, thought form, thought content (e.g. delusions), and perceptual disturbances (e.g. hallucinations) [28]. The variation in presentation that this creates may explain the difficulty staff experience in diagnosing delirium, and the many psychiatric differentials including depression, psychosis and dementia.

Hypoactive delirium subtype, being an input variable in the predictive model, also reinforces the need for education in the different clinical presentations of delirium. Unlike patients with a hyperactive presentation who quickly attract attention from nursing and medical staff, those with hypoactive symptoms are more likely to stay under the radar and their symptoms are often misconstrued as symptoms of depression [7, 11]. Our finding of patient’s age as an input variable with lesser importance for the predictive model is not consistent with the literature [6, 28]. Otani et al. [14] also found age to be a non-significant risk factor, suggesting that this may not be a true association. Elderly patients often present with hypoactive delirium, and psychiatric conditions such as depression and dementia are highly prevalent in the elderly population. It is possible that these factors may confound the high rates of misdiagnosis seen in elderly patients in previous studies; repeated research in a larger sample is necessitated to further explore this association.

Pain as a primary symptom has been shown to be an important factor previously [3, 10, 13] and may relate to diagnostic overshadowing, whereby symptoms of delirium are misconstrued as responses to pain. In our study, pain importance, as input variable for the predictive model, was low. Only 25 patients in the sample (34%) had pain as a major symptom. The importance we found between referral unit and delirium diagnosis was low. However, Agbayewa [22] found that patients on medical units were more likely to attract accurate diagnoses. This may relate to medical staff being able to spend more time with their patients than surgical teams. Time is an important factor in identifying and accurately diagnosing delirium; thus, it is unsurprising that in an emergency setting the misdiagnosis rate was 76% [19].

Continuity of care is imperative in managing patients with delirium, and staff with only brief periods of contact may be misled by fluctuations in these patients’ presentations. The same patient can, in the morning, exchange pleasantries with the staff and in the afternoon be markedly disoriented and cognitively impaired [29]. Spending more time with patients, reassessing their charts and speaking to family members may ameliorate this pitfall, though all of these measures take time, a scarce commodity in acute clinical settings [30]. Evidence from surveys and workshops has shown that lack of time for assessment in acute settings contributes to low detection rates of delirium [31]. Zou et al. [30] even showed that a nurse clinician’s diagnosis, using a structured delirium diagnostic instrument, with more time and multiple occasions to review patients, was more accurate than a diagnosis by psychiatrists. Our findings also indicated that having a 4AT score completed prior to referral was a relatively important variable of the predictive model for an accurate diagnosis. A sound evidence base supports screening instruments for delirium, and studies have shown repeatedly that without instruments, delirium is often missed [23, 24, 31]. Despite this, nearly half of the patients in our sample did not have a 4AT completed. This highlights the need for having these tools readily available on wards and, most importantly, educating medical and allied health students and junior staff on the use and benefits of these tools so they become integrated into commonplace medical care in the future.

The length of hospital stay was negligible factor (OR 1.00) in this study, which is at odds with previous studies [32]. This may be explained by a large number of our accurately diagnosed patients having severe burns, leading to longer stays regardless of delirium resolution. Additionally, there are many factors affecting length of stay in this cohort, and in any cohort, so that modifying one variable may be unlikely to produce a large difference between groups. Morbidity and mortality are associated with delirium and worse outcomes if treatment is delayed [1, 6, 32, 33]. Heymann et al. found delayed treatment of delirium predicted increased mortality in ICU patients compared with immediate treatment [33]. Patients whose treatment was delayed by 24 h were more likely to develop nosocomial infections or be mechanically ventilated, suggesting that not only the presence of delirium, but how quickly an accurate diagnosis is made, can affect morbidity, length of stay, hospital costs and mortality. This may be in part due to delirium increasing the likelihood of delay or failure to treat underlying conditions with serious consequences including worsening medical illness, self-injury and death [10, 28]. Treatment for presumed psychiatric illness can also worsen delirium, such as the use of anticholinergic antidepressants or antipsychotics [11].

### Limitations

This study had several limitations, including a relatively small sample size, which limited the predictive power of the classifier algorithm. As this study was retrospective and derived from the electronic clinical record, we were limited to the information available. A range of CLP clinicians diagnosed delirium and inter-individual variation is likely, although clinical diagnosis was made against DSM 5 criteria and discussed at the daily multidisciplinary team meeting including a consultant psychiatrist. Evidence of pain as a primary symptom, delirium subtype and other factors were sourced from the medical record; some cases may have been missed using this method. Cases of delirium were limited to those referred to CLP and many other cases of delirium in the hospital may have been undetected or misdiagnosed. Length of stay and mortality data were collected on a census date only a matter of days after completion of the study period, so these figures may have been underestimated. We did not look at characteristics of referring physicians, which may enrich future studies assessing misdiagnosis of delirium. Despite these limitations, using a machine learning approach to predict delirium misdiagnosis is novel. The Machine Learning-Logistic regression model presented in this study is useful for clinical decision-making as well as for quality control, and future work can implement this algorithm for further validation.

## Conclusions

Our findings indicate that delirium is commonly misdiagnosed. We propose a predictive classifier model to accurately diagnose delirium in hospital setting. Patient’s psychiatric history is strongly associated with misdiagnosis. There is an urgent need for education of non-psychiatric staff [2, 26]. Education should highlight pitfalls such as misinterpretation of a past psychiatric history, and clarify clinical features of hypoactive delirium and the presentation of delirium in the elderly. Importantly, education should also encourage use of delirium screening instruments wherever possible.

## Data Availability

The datasets used and/or analyzed during the current study are available from the corresponding author on reasonable request.

## Abbreviations

CLP: Consulation-Liason Psychiatry
OR: Odds Ratio
